# The Increase in Childhood Obesity and Its Association with Hypertension during Pandemics

**DOI:** 10.3390/jcm12185909

**Published:** 2023-09-12

**Authors:** Corina Maria Vasile, Paul Padovani, Stefan Dumitrache Rujinski, Dragos Nicolosu, Claudia Toma, Adina Andreea Turcu, Ramona Cioboata

**Affiliations:** 1 Department of Pediatric and Adult Congenital Cardiology, University Hospital of Bordeaux, F-33600 Bordeaux, France; corinva.vasile93@gmail.com; 2 Nantes Université, CHU Nantes, Department of Pediatric Cardiology and Pediatric Cardiac Surgery, FHU PreciCare, F-44000 Nantes, France; padovanipaul17@gmail.com; 3 Nantes Université, CHU Nantes, INSERM, CIC FEA 1413, F-44000 Nantes, France; 4 Pneumology Department, University of Medicine Carol Davila, 020021 Bucharest, Romania; srujinski@yahoo.com; 5 Pneumology Department, Victor Babes University Hospital Craiova, 200515 Craiova, Romania; nicolosud@yahoo.com (D.N.); ramona_cioboata@yahoo.com (R.C.); 6 Faculty of Dentistry, University of Pharmacy and Medicine Craiova, 200349 Craiova, Romania; 7 Pneumology Department, University of Medicine and Pharmacy, 200349 Craiova, Romania

**Keywords:** obesity, hypertension, pandemics, childhood, food habits, sedentary

## Abstract

There has been a major ongoing health impact of the COVID-19 pandemic on children’s lives, including lifestyle and overall health. Enforcement of prevention measures, such as school closures and social distancing, has significantly affected children’s daily routines and activities. This perspective manuscript aims to explore the rise in childhood obesity and its association with hypertension during pandemics. The COVID-19 pandemic has led to significant disruptions in children’s routines, including reduced physical activity, increased sedentary behavior, and changes in dietary patterns. These factors, coupled with the psychological impact of the pandemic, have contributed to an alarming increase in childhood obesity rates. This paper has highlighted the concerning increase in childhood obesity and hypertension during pandemics. The disruptions caused by the COVID-19 pandemic, including reduced physical activity, increased sedentary behaviors, and changes in dietary patterns, have contributed to the rise in these health conditions. It is crucial to recognize the long-term consequences of childhood obesity and hypertension and the urgent need for a comprehensive approach to address them.

## 1. Introduction

There has been a major ongoing health impact of the COVID-19 pandemic on children’s lives, including lifestyle and overall health. Enforcement of prevention measures, such as school closures and social distancing, has significantly affected children’s daily routines and activities. Consequently, children experienced significant changes in physical activity levels, inactivity habits, and eating disorders [1,2,3].

Childhood obesity is a major public health concern characterized by abnormal or excessive adipose tissue accumulation. It occurs when there is a chronic imbalance between caloric intake and caloric consumption, resulting in the storage of excess energy in adipose tissue. It is associated with various adverse health outcomes, including hypertension. Hypertension, or high blood pressure, is defined as having three elevated systolic or diastolic blood pressure (BP) readings for the subject’s age, height, and sex, which can lead to long-term cardiovascular complications [4,5].

The COVID-19 pandemic has created unique circumstances contributing to the increased prevalence of obesity and subsequent hypertension among children. Disruption of children’s daily routines, limited access to physical activity, increased sedentary lifestyles, and changes in dietary habits have all increased obesity rates in children. Moreover, the psychological impact of the pandemic, including increased stress levels and emotional eating, has further amplified this phenomenon [6,7].

Understanding the relationship between pandemics and the increased prevalence of childhood obesity and hypertension is crucial for several reasons. The most important is it sheds light on the specific factors contributing to the rise in these conditions during times of crisis.

This knowledge can inform targeted interventions and public health strategies to mitigate the impact and prevent long-term consequences. Then, recognizing the link between pandemics, childhood obesity, and hypertension highlights the need for a comprehensive approach that addresses children’s physical and mental well-being during crises.

By examining this relationship, healthcare professionals, policymakers, educators, and families can collaborate to implement effective prevention and intervention measures to protect the health and well-being of children [8].

This narrative review aims to highlight the important problem of childhood obesity and its relationship to hypertension during the pandemic. By systematically reviewing the existing literature and synthesizing the scientific evidence, this review aims to explore whether childhood obesity rates have increased under pandemic conditions and to elucidate the relationship between childhood obesity and the increased risk of developing hypertension in this context. In addition, the aim was to identify the factors underlying this association, whether lifestyle changes, dietary habits, or other pandemic-related stressors. Ultimately, the findings of this analysis aim to provide valuable insights into the complex interaction between pandemics, childhood obesity, and hypertension, providing a comprehensive perspective on the broader public health implications and potential prevention and intervention strategies.

## 2. Methodology

This narrative review aims to provide insight into the increasingly serious problem of childhood obesity and its complex connection to hypertension, particularly in the context of the pandemic. A systematic approach to searching relevant databases, including PubMed, Embase, and Scopus, was meticulously performed using adapted search terms relating to childhood obesity, hypertension, and the COVID-19 pandemic. Our approach was complemented by handsearching of peer-reviewed journals and meticulous examination of reference lists in fundamental articles. To reduce potential selection bias, the eligibility of each reference was assessed diligently by two separate authors, and any discrepancies were resolved by consensus. Inclusion criteria included studies and sources published to date, with no language restrictions encompassing a diverse literature range. Crucially, it is essential to recognize that this narrative review, by design, focuses on synthesizing existing knowledge and does not formally assess the quality of individual studies, as its primary purpose is to comprehensively expose the complex interplay between childhood obesity, hypertension, and pandemic issues. This methodology outlines our commitment to provide a comprehensive, informative, and contextually relevant presentation of this challenging topic.

## 3. The Well-Established Connection between Childhood Obesity and Hypertension Long before the COVID-19 Pandemic

The complex interaction between childhood obesity and hypertension has been an important research topic even before the COVID-19 pandemic. This pattern of conclusive association underscores the profound implications of overweight on blood pressure, highlighting the imperative need for preventive interventions and targeted prevention strategies. Consistent research over the years has highlighted the lasting impact of childhood obesity on susceptibility to hypertension, necessitating a comprehensive understanding of this relationship in the pediatric context.

Statistical analyses have consistently revealed a notable association between higher body mass index (BMI) and increased blood pressure levels. In 2008, Salvadori et al. [9] conducted a study within a rural Canadian community, examining a cohort of 675 children. The findings revealed that 11.4% of the children were classified as obese, while 18.1% were categorized as overweight. Notably, both obesity and overweight were correlated with elevated blood pressure.

In another study conducted by Kovacs et al. [10], in 2010, the prevalence of abdominal obesity was investigated across varying weight categories in a cohort of children. Among normal-weight children, the prevalence of abdominal obesity was 3.7%, while in overweight and obese children, it surged to 51.7% and 89.9%, respectively. The study also examined systolic and diastolic blood pressure (SBP and DBP) in abdominal obesity. Notably, children with abdominal obesity exhibited a higher SBP and DBP than those with a normal waist circumference (WC), a trend observed across both normal and overweight body mass index (BMI) categories. Even after accounting for age and BMI, WC demonstrated significant correlations with SBP and DBP across all BMI categories. Despite these findings, the study did not yield significant odds ratios (ORs) for prehypertension or hypertension associated with abdominal obesity in the normal-weight category. However, among overweight children, the prevalence of prehypertension and hypertension was notably higher among those with abdominal obesity, as indicated by ORs of 1.42 and 1.35, respectively. A similar trend was observed among obese children, with prehypertension being almost two times higher in those with abdominal obesity (22.5% vs. 11.8%), although not yielding a significant OR.

Mehad et al. [11] conducted a study focusing on Moroccan adolescents to assess the prevalence of hypertension and its correlation with body fat mass (BFM), body mass index (BMI), and waist circumference (WC). Within a participant pool of 167 adolescents aged 11–17 years, 29.3% were classified as overweight and 12.6% as obese. The study revealed hypertension (HT) and prehypertension (pre-HT) rates of 17.4% and 9.6%, respectively, with boys and overweight–obese groups displaying a higher prevalence. Notably, overweight–obese participants exhibited significantly elevated systolic and diastolic blood pressure (SBP, DBP) compared to their healthy-weight counterparts. Associations between SBP, DBP, BMI, WC, and BFM were significant, especially pronounced in girls, highlighting an elevated hypertension risk with increasing BMI levels.

In a study led by Meydanlioglu A. et al. [12], in Antalya, Turkey, the objective was to investigate the prevalence of obesity and hypertension in school-age children while identifying correlated risk factors. The study encompassed 5160 students aged 5 to 15 years across 21 schools. The results highlighted that among the participants, 11.4% were classified as overweight, 11.8% as obese, 6.8% exhibited prehypertension, and 5.7% displayed hypertension. The investigation also unveiled specific factors associated with these conditions. Overweight and obesity were linked to the school level, location, and the father’s educational status. In contrast, hypertension was associated with BMI, school level, location, and the mother’s working status. Noteworthy, the research emphasized a notably higher prevalence of hypertension, overweight, and obesity among children residing in rural areas.

Another important study was the one conducted by Duarte Rebelo [13], which aimed to assess how different markers of obesity relate to blood pressure (BP) in Portuguese children and adolescents. The study included 2494 boys and 2589 girls. Importantly, the study underscored that overweight and obesity were associated with a substantially increased risk of hypertension. Overweight boys had an odds ratio of 2.26 for hypertension, while obese boys had an even higher odds ratio of 3.36. Similarly, overweight girls had an odds ratio of 1.58, and obese girls had an odds ratio of 2.31 for hypertension. In conclusion, this study showed that BMI, rather than BF or waist measurements, demonstrated consistent and independent associations with BP levels in children and adolescents. Furthermore, it highlighted the significant role of overweight and obesity in elevating this population’s hypertension risk.

A more extensive study, conducted from February to December 2009 in Shanghai, involved 78,114 participants and aimed to investigate the connection between hypertension prevalence and overweight/obesity among Chinese children and adolescents [14]. Based on the results of this study, the prevalence of hypertension was notably higher in overweight and obese children compared to those of normal weight. In conclusion, this large-scale study from Shanghai underscored the connection between BMI, waist circumference (WC), and blood pressure levels. Being overweight and obese significantly increases the hypertension risk in Chinese children and adolescents, with WC emerging as a more sensitive indicator than BMI.

In 2014, Dulskiene et al. [15] conducted a Lithuanian study involving 7457 adolescents aged 12–15 years. Their study aimed to investigate the relationships between overweight, obesity, abdominal obesity, prehypertension, and hypertension among this age group in Lithuania. The study’s findings highlighted a noteworthy high blood pressure (BP) prevalence among Lithuanian adolescents aged 12–15.

These studies support the well-established relationship between childhood obesity and high blood pressure, a connection observed consistently before the COVID-19 pandemic. Evidence from various studies in different regions shows that a higher body mass index (BMI), abdominal obesity, and increased body fat are consistently associated with higher blood pressure levels in young people. These findings highlight the lasting impact of childhood obesity on the risk of hypertension, underlining the urgent need for effective preventive measures and specific strategies to combat childhood obesity. Recognizing and addressing this complex interaction between childhood obesity and hypertension is crucial to protect the health of young people and develop effective public health interventions.

## 4. Trends in Childhood Obesity and Hypertension Amidst the Pandemic

Several studies have indicated a notable surge in cases of obesity and its associated comorbidities during the COVID-19 pandemic. The prevalence of obesity has witnessed a significant increase compared to the pre-2019 period, largely attributed to alterations in the dietary habits of children and adolescents. The pandemic-induced changes in food availability and consumption patterns have emerged as pivotal contributors to disruptions in the holistic well-being of young individuals. Children from poor backgrounds, who rely on school-provided meals for proper and well-balanced nutrition, faced substantial challenges due to lockdown measures. The closure of schools disrupted this crucial source of sustenance, exacerbating the risk of physical, mental, and social health imbalances [16].

In India, a comprehensive study conducted during the pandemic unveiled a distressing deterioration in eating behaviors, with many children resorting to unhealthy snacking and excessive food consumption [17]. The closure of schools not only curtailed physical activity but also profoundly impacted children’s mental health.

The shift to online learning led to a considerable rise in sedentary behavior, with children spending prolonged hours in front of screens. The reduction in outdoor activities, physical exercise, and social engagements further intensified the challenges. The amalgamation of reduced physical exertion, limited social interactions, apprehensions related to canceled examinations, and social distancing contributed to heightened stress and depression. These psychological factors could trigger overeating and subsequent obesity, fostering decreased social interactions and long-term self-esteem issues.

The past 3 years have witnessed a significant surge in obesity cases, particularly during the pandemic. This escalation in obesity rates has given rise to a spectrum of associated complications. The pronounced increase in obesity, particularly among children and adolescents, can be attributed to a combination of factors, including reduced physical activity, heightened stress levels, excessive eating, and the widespread implementation of global lockdowns. The imposition of home isolation measures, the closure of schools, and the prevalence of online classes have collectively contributed to a lifestyle characterized by diminished physical engagement and prolonged periods of indoor confinement [18].

Qiu’s study examined potential sex differences in BMI and blood pressure among Chinese school-age children amidst lifestyle changes, particularly during the COVID-19 quarantine. This cohort study, encompassing 445 children, investigated BMI and blood pressure alterations over five months. The study aimed to identify predictors of overweight, obesity, and elevated blood pressure (EBP). The results showed an increase in the proportion of boys with overweight and obesity (*p* = 0.036) and a rise in pre-EBP and EBP for both genders (*p* = 0.004 in boys; *p* < 0.001 in girls) during the quarantine [19].

The COVID-19 pandemic has led to a disturbing increase in obesity and related health issues, particularly among children and adolescents. This increase in obesity rates can be attributed to several factors, including disrupted eating habits, reduced physical activity, and prolonged sedentary behavior due to shutdowns and the shift to online learning. Children from disadvantaged backgrounds, who relied on school-provided meals for a balanced diet, faced additional challenges. The impact of the pandemic on mental health, including increased stress and anxiety, also played a role in driving unhealthy eating behaviors. As a result, there has been a significant increase in obesity in recent years, which calls for a special and urgent approach to maintaining young people’s physical and psychological well-being during and after the pandemic.

## 5. Factors Contributing to Childhood Obesity during Pandemics

During pandemics, several factors contribute to the increased prevalence of childhood obesity. Firstly, the closures of schools and implementation of social distancing measures have reduced children’s physical activity opportunities. With limited access to structured physical education classes, sports, and recreational facilities, children have experienced a decrease in their overall activity levels. Secondly, the transition to remote learning and increased reliance on screens have increased sedentary behavior among children. Prolonged screen time for virtual classes, entertainment, and socialization has reduced time for physical activity and outdoor play [20,21,22].

**Figure 1:**
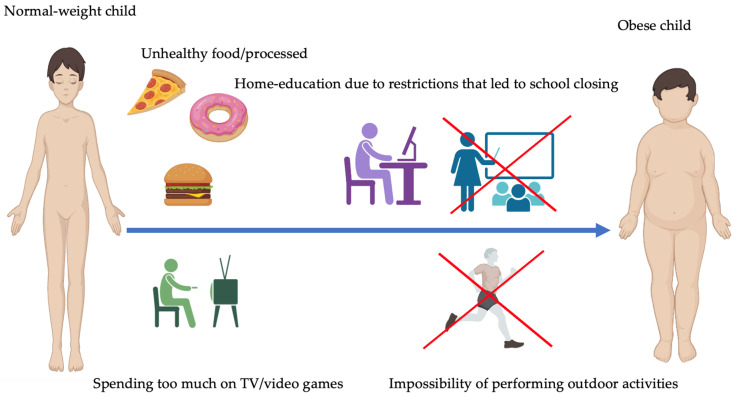
The most important factors contributing to childhood obesity during pandemics.

Additionally, the restrictions on outdoor activities and limited opportunities for active play have further exacerbated sedentary behaviors. Lastly, changes in dietary patterns have played a role in the rise in childhood obesity during pandemics. With disrupted routines and increased stress, there has been a shift toward consuming processed foods high in unhealthy fats, sugars, and calories. This and decreased access to nutritious meals have contributed to an imbalanced diet. The combination of reduced physical activity, increased sedentary behaviors, and unhealthy dietary habits during pandemics has created an environment conducive to the development of childhood obesity. Addressing these factors is crucial to prevent and manage childhood obesity during crises [23,24,25,26]. Figure 1 presents some of the most important factors contributing to childhood obesity during the COVID-19 pandemic.

Childhood obesity during pandemics is driven by reduced physical activity (due to school closures and social distancing), increased sedentary lifestyles (caused by distance learning and excessive screen time), limited opportunities for active play, and changes in eating habits that promote processed high-calorie foods. Addressing these factors is crucial in managing childhood obesity during pandemics.

## 6. Psychological Impact of Pandemics on Childhood Obesity

Pandemics have significant psychological effects on children, which can contribute to developing or exacerbating childhood obesity. The heightened stress and anxiety associated with the uncertainty and disruptions caused by pandemics can impact children’s mental well-being and eating behaviors. Social isolation resulting from lockdowns and limited peer interaction can lead to feelings of loneliness and emotional distress, which may increase the risk of emotional eating as a coping mechanism. Disrupted routines, including sleep and meal schedule changes, can contribute to irregular eating patterns and unhealthy food choices. Moreover, the absence of social support systems and reduced access to mental health resources during pandemics can hinder the management of stress and emotional well-being, potentially exacerbating the risk of childhood obesity. Recognizing and addressing the psychological impact of pandemics on children is essential in providing appropriate support, promoting healthy coping mechanisms, and mitigating the risk of childhood obesity [27,28,29,30,31].

The psychological impact of pandemics on childhood obesity extends beyond stress, anxiety, and emotional eating. Social isolation and disrupted routines can profoundly affect children’s mental well-being and eating behaviors. Social connections and peer interactions play a crucial role in children’s development, and the absence of these interactions during periods of isolation can lead to feelings of loneliness, sadness, and decreased self-esteem. This emotional distress may contribute to unhealthy coping mechanisms, such as seeking comfort in food or eating emotionally [32,33,34].

Furthermore, the disruption of daily routines, including school closures and changes in family dynamics, can impact children’s eating patterns. With less structured schedules, mealtimes may become irregular, leading to snacking or inconsistent eating habits. The availability of food at home, combined with boredom and emotional triggers, can increase the likelihood of consuming high-calorie, low-nutrient foods. Limited access to fresh and nutritious meals and reliance on processed and convenience foods can further exacerbate unhealthy eating habits during periods of crisis [35,36,37].

The psychological impact of pandemics on childhood obesity is a complex interplay between emotional well-being, social connections, and disrupted routines. Addressing these factors through supportive interventions that prioritize mental health, promote healthy coping strategies, and provide guidance on maintaining regular eating patterns and making nutritious food choices is crucial. Creating a supportive environment that addresses the emotional needs of children, fosters social connections, and emphasizes healthy lifestyles can help mitigate the risk of childhood obesity during pandemics [38,39,40].

## 7. Association between Childhood Obesity and Hypertension

The association between childhood obesity and the development of hypertension is well established and understanding the mechanisms underlying this relationship is crucial in addressing both conditions. Obesity in children refers to excessive body fat accumulation, which can profoundly affect various physiological systems, including the cardiovascular system [41,42].

The connection between childhood obesity and hypertension can be attributed to several mechanisms. One important factor is insulin resistance, commonly observed in obese individuals. Insulin resistance refers to the body’s decreased ability to respond to the hormone insulin, leading to elevated insulin levels in the bloodstream. Insulin resistance promotes sodium and water retention and increases the production of vasoconstrictive substances, contributing to increased blood pressure [43,44,45].

Inflammation is another mechanism linking childhood obesity and hypertension. Adipose tissue in obese individuals releases proinflammatory molecules called adipokines, leading to chronic low-grade inflammation. This chronic inflammation disrupts the normal functioning of blood vessels, promoting vasoconstriction and impairing their ability to relax, contributing to elevated blood pressure [46,47,48].

Additionally, obesity-related vascular dysfunction plays a role in the development of hypertension. The excess body fat in obese children leads to endothelial dysfunction, characterized by the impaired function of the cells lining the blood vessels. Endothelial dysfunction reduces the production of nitric oxide, a substance that helps dilate blood vessels, and increases the production of vasoconstrictive factors, contributing to elevated blood pressure [48,49,50,51].

Moreover, other factors associated with childhood obesity, such as dyslipidemia (abnormal lipid levels) and increased sympathetic nervous system activity, further contribute to the development of hypertension. Dyslipidemia is characterized by elevated levels of triglycerides and low-density lipoprotein cholesterol, which can lead to atherosclerosis and impaired blood vessel function. Increased sympathetic nervous system activity can lead to a higher heart rate and blood pressure, contributing to hypertension [42,43,44,45,52,53,54,55].

**Figure 2:**
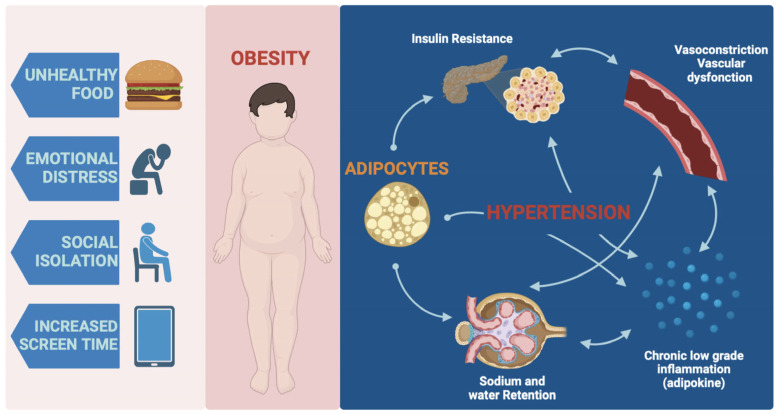
Obesity-induced hypertension in children after pandemics.

The primary goal of competently addressing these complex issues is to ameliorate risk factors and promote sustainable cardiovascular well-being in the pediatric cohort [56,57]. Figure 2 shows the relationship between risk factors and obesity during the pandemic period and the underlying mechanisms that led to the associated hypertension.

## 8. Underlying Genetics of Childhood Obesity

Genetic predisposition plays a significant role in the development of both obesity and hypertension in childhood. While lifestyle factors contribute, understanding the genetic underpinnings is crucial in effectively addressing these health concerns.

Obesity is a multifaceted and intricate disorder influenced by genetic and environmental components. The surge in obesity prevalence is notably shaped by environmental factors—specifically, the prevalence of calorie-dense food sources coupled with sedentary lifestyles that curtail energy expenditure. Recognizing the interplay between obesity and hereditary factors has been constrained by a constrained comprehension of genetics at the human genome level and an incomplete understanding of the intricate biological pathways underpinning obesity [58].

The significance of genetic factors in obesity has been established through twin and family studies. A positive familial history of obesity increases the risk of childhood obesity. Among monozygotic (identical) twins, a notably higher concordance rate for obesity is observed compared to dizygotic (nonidentical) twins. Within the realm of twin studies, the heritability of obesity ranges from 40% to 75% [59].

The genetics underlying obesity may be categorized into two main groups: syndromic and nonsyndromic obesity, which may or may not be accompanied by congenital anomalies and developmental delay. Certain conditions such as Prader–Willi, fragile X, Bardet–Biedl, Cohen, and Albright hereditary osteodystrophy (AHO) syndromes manifest with developmental delay and early-onset obesity [60]. These syndromes manifest with cognitive impairment, distinctive physical features, and specific developmental abnormalities in various organs [61]. Recognizing the interplay of obesity, developmental delay, dysmorphic components, and organ dysfunction is crucial, prompting the need for genetic referrals to facilitate comprehensive evaluation. On the other hand, nonsyndromic obesity can be further classified as monogenic, polygenic, or chromosomal in origin. Monogenic obesity results from variants in individual genes, whereas polygenic obesity involves the participation of multiple genes, often from gene families. The latter group may or may not present syndromic features, but obesity and distinct phenotypes are consistently observed.

Traditionally, the classification of obesity has encompassed two primary categories: firstly, monogenic obesity—exhibiting Mendelian inheritance—tends to be infrequent, severe, and early-onset. It ensues from either minuscule or substantial chromosomal deletions, along with defects in single genes. In contrast, polygenic obesity, also recognized as common obesity, emerges from the cumulative influence of numerous polymorphisms, each exerting a modest impact. This form of obesity adheres to a heritability pattern akin to other intricate traits and conditions [62].

Despite their apparent dichotomy, studies on gene discovery for monogenic and polygenic obesity have coalesced around a shared underlying biology. Notably, the central nervous system (CNS) and the intricate neural pathways governing the pleasurable facets of food intake have emerged as pivotal determinants of body weight in monogenic and polygenic obesity. Furthermore, preliminary evidence suggests a potential interplay wherein the expression of mutations responsible for monogenic obesity could be modulated—albeit partially—by an individual’s polygenic predisposition to obesity [63].

In the initial phases of gene discovery concerning monogenic obesity, the investigative approach primarily centered around a case-centric design. This methodology involved a meticulous examination of individuals grappling with severe obesity alongside both affected and unaffected family members. The primary objective was pinpointing potential causal mutations disrupting specific genes, employing techniques such as Sanger sequencing.

In stark contrast, the exploration of genetic variations associated with common obesity has been undertaken on a more expansive scale through large-scale population studies. These investigations have employed either a case-control framework or have delved into continuous traits like BMI. Originally, gene discovery endeavors for both forms of obesity were guided by specific hypotheses, focusing on a predetermined set of candidate genes believed to be involved in the intricate regulation of body weight.

Over the past two decades, a significant paradigm shift has occurred, propelled by advancing high-throughput genome-wide genotyping and sequencing technologies. Bolstered by a comprehensive understanding of human genetic architecture, researchers now possess the capabilities to conduct exhaustive analyses of genetic variants across the entire genome. This evolution has enabled a more hypothesis-generating approach, empowering the comprehensive exploration of the multifaceted role that genetic factors play in the intricate regulation of body weight [62].

While monogenic obesity is rare, it results from gene mutations within the leptin/melanocortin pathway in the hypothalamus. This pathway regulates food intake, satiety, body weight, and energy metabolism [64]. Leptin, a key player in this pathway, not only governs eating behaviors but also influences the onset of puberty and T-cell immunity [65].

Leptin, a protein secreted by white adipose tissue, is encoded by a gene located on chromosome 7 in humans. This protein traverses the blood–brain barrier to engage with presynaptic GABAergic neurons within the hypothalamus. Its action culminates in suppressing appetite and elevating energy expenditure, thereby influencing weight regulation [66].

Although the role of leptin and the associated leptin receptor gene in human obesity is progressively unraveling, it remains enigmatic and requires further exploration [67]. Noteworthy is a study by Farooqi et al. [60], wherein inherited human leptin deficiency yielded severe early-onset obesity, marked by instances such as 8 years and 86 kg or 2 years and 29 kg. This obesity was traced back to a homozygous obesity leptin gene with a frame-shift mutation (deletion of G133), synthesizing a truncated protein [60].

In tandem, other investigations unveiled a paradox in obese patients, revealing elevated leptin levels but a simultaneous decrease in soluble leptin receptors. This intricate relationship directly influences leptin function. Substantiating this, a study involving 110 participants—comprising 55 obese individuals and 55 healthy controls—highlighted markedly higher leptin levels in the obese cohort than in the controls [68]. This interplay is recognized as leptin resistance.

These leptin receptors, distributed within the central nervous system and across peripheral organs, including the liver, skeletal muscles, pancreatic beta cells, and adipose cells, collectively contribute to intricate energy regulation mechanisms.

Approximately 3% of obese children exhibit mutations in the leptin (LEP) gene and the leptin receptor (LEPR), which can lead to delayed puberty and immune dysfunction [65,69]. Other mutations within the leptin–melanocortin pathway, such as in proopiomelanocortin (POMC), melanocortin receptor 4 (MC4R), brain-derived neurotrophic factor (BDNF), and the tyrosine kinase receptor B (NTRK2) genes, can also contribute to obesity [70,71]. The presentation of monogenic forms typically occurs in early childhood, often by age 2, and is characterized by severe obesity and atypical feeding behaviors [72].

Polygenic obesity is the prevalent form of obesity, arising from the cumulative impact of numerous genetic variations. This condition emerges from the intricate interplay between genetic predisposition and environmental influences, a phenomenon termed gene–environment interaction (GEI) [73,74,75,76].

Notably, genome-wide association studies (GWAS) have pinpointed specific gene variants, often referred to as single nucleotide polymorphisms (SNPs), associated with body mass index (BMI). These variants likely collaborate synergistically, collectively influencing body weight [77]. Within various genes, researchers have uncovered genetic variants contributing to heightened hunger and increased food consumption, potentially leading to excessive weight gain [78,79,80].

In cases where an individual’s genetic makeup confers susceptibility to obesity, exposure to an environment conducive to weight gain can lead to an energy imbalance. This imbalance is driven by behaviors that favor energy conservation over expenditure [81]. The research underscores that individuals with obesity-associated genetic variations might exhibit tendencies such as heightened food intake, reduced physical activity, diminished metabolism, and an inclination toward storing body fat [75,78,79,81,82].

In recent times, the role of epigenetic factors in the genesis of obesity has come to the forefront [83]. Epigenetics involves modifying gene expression without altering the underlying DNA sequence. This process includes adding chemical tags (methyl groups) to an individual’s chromosomes. These modifications can govern the activation and deactivation of pivotal genes. During infancy, intricate physiological and psychological adaptations unfold, potentially shaping the trajectory toward health or illness.

The concept of Developmental Origins of Health and Disease (DOHaD) underscores how the early-life environment can imprint an enduring influence on the risk of chronic ailments later in life. This imprinting, facilitated by epigenetic changes, arises from fetal programming. Maternal nutrition during prenatal or early postnatal stages can potentially trigger these epigenetic modifications, heightening the susceptibility to chronic conditions like obesity, metabolic disorders, and cardiovascular diseases. These epigenetic alterations may persist, exerting intergenerational effects on the health of both offspring and adults [71,84,85,86].

Likewise, adverse childhood experiences (ACEs) have been associated with a wide spectrum of unfavorable outcomes mediated through epigenetic mechanisms [87]. These experiences can foster unhealthy eating behaviors [88,89]. Moreover, factors including diet, physical activity, and environmental and psychosocial stressors can induce epigenetic modifications, augmenting the vulnerability to weight gain [90].

## 9. Implications for Public Health and Interventions

The public health consequences of childhood obesity and hypertension during the pandemic are important. Today’s global health challenge requires urgent action to address these issues and reduce the long-term effects [48,49,91,92].

Healthcare providers are responsible for educating families and raising awareness about the impact of pandemics on children’s health. They can guide healthy lifestyle behaviors, including promoting balanced nutrition, increasing physical activity, and monitoring blood pressure. Healthcare providers can also conduct regular screenings and assessments to identify children at risk for obesity and hypertension, enabling early intervention and personalized care [48,50,93,94].

Schools are essential partners in public health efforts. They can implement evidence-based programs that promote healthy eating habits, provide nutritious meals, and incorporate physical activity into the curriculum. Schools should also prioritize mental health support for students, as psychological well-being is closely linked to obesity and hypertension. Collaborating with healthcare providers and families, schools can create an environment that fosters healthy behaviors and supports the overall well-being of children [52,95].

Political authorities play a crucial role in determining the environment in which children live and grow up. They can adopt policies prioritizing access to healthy food, regulating the marketing of unhealthy products, and setting standards for physical education and recreational facilities. Policymakers should also allocate resources for research, surveillance, and the implementation of effective prevention and intervention programs. Creating a supportive policy framework can enable positive changes that promote healthy lifestyles for children during pandemics and beyond [53,54,96,97].

Families play a central role in preventing childhood obesity and hypertension. They can create a home environment that supports healthy eating habits by providing nutritious meals, limiting the availability of processed foods, and promoting family meals. Families can also encourage physical activity by engaging in active play, limiting screen time, and fostering a positive relationship with exercise. Additionally, addressing the psychological well-being of children by creating a supportive and nurturing environment is vital [98,99,100].

Interventions to address childhood obesity and hypertension during pandemics should take a comprehensive approach. This includes promoting healthy eating habits through nutrition education, increasing access to affordable and nutritious foods, and implementing school-based interventions. Encouraging physical activity opportunities through active play, sports programs, and community initiatives is crucial. Moreover, addressing the psychological well-being of children through mental health support, stress management strategies, and promoting resilience is essential [101,102].

In conclusion, public health initiatives are urgently needed to address childhood obesity and hypertension during pandemics. Healthcare providers, schools, policymakers, and families must work together to implement comprehensive prevention and intervention strategies. By promoting healthy eating habits, increasing physical activity opportunities, and addressing psychological well-being, we can mitigate the impact of pandemics on children’s health and foster a generation of healthier individuals. Collaborative efforts and a multisectoral approach are key to creating an environment supporting healthy lifestyles and reducing childhood obesity and hypertension [103,104,105,106].

## 10. Future Directions and Research Needs

Future research in childhood obesity and hypertension during pandemics should focus on several areas to enhance our understanding and improve interventions. Longitudinal studies are needed to assess the long-term effects of the pandemic on the prevalence and trajectory of childhood obesity and hypertension. These studies will provide insights into the lasting impact of pandemic-related disruptions on children’s health and help identify potential strategies for prevention and intervention.

Evaluating the effectiveness of interventions targeting childhood obesity and hypertension within the context of pandemics is crucial. Research should investigate the outcomes of various interventions, such as school-based programs, community initiatives, and digital health interventions, to determine their effectiveness in mitigating the risk and managing these conditions during times of crisis. Understanding the most effective strategies will inform evidence-based recommendations for future public health interventions.

Exploring innovative approaches, such as telehealth and digital interventions, is essential to reach children and families during times of crisis. Investigating the feasibility, acceptability, and effectiveness of remote interventions, including telehealth consultations, mobile applications, and virtual support groups, can provide alternative means of delivering healthcare services and support to children and families when traditional in-person interactions are limited. These digital approaches can potentially overcome barriers to access and engage children and families in managing obesity and hypertension during pandemics and beyond.

Incorporating interdisciplinary collaborations is also crucial for future research. Collaboration among healthcare providers, researchers, policymakers, educators, and families will enable a comprehensive approach to addressing childhood obesity and hypertension during pandemics. By working together, stakeholders can develop and implement strategies encompassing nutrition, physical activity, mental health, and policy changes to promote healthy lifestyles and improve outcomes for children.

To sum up, future research in childhood obesity and hypertension during pandemics should include longitudinal studies to assess long-term effects, evaluations of intervention effectiveness, and exploration of innovative approaches. By addressing these research needs, we can enhance our understanding of the impact of pandemics on children’s health and well-being and develop evidence-based strategies to prevent and manage childhood obesity and hypertension in crises.

## 11. Conclusions

In conclusion, this paper has highlighted the concerning increase in childhood obesity and hypertension during pandemics. The disruptions caused by the COVID-19 pandemic, including reduced physical activity, increased sedentary behaviors, and changes in dietary patterns, have contributed to the rise in these health conditions. It is crucial to recognize the long-term consequences of childhood obesity and hypertension and the urgent need for a comprehensive approach to address them. Collaborative efforts are required from healthcare professionals, policymakers, educators, and families to prioritize the health and well-being of children in crisis situations. This entails implementing evidence-based interventions, promoting healthy eating habits, increasing physical activity opportunities, addressing psychological well-being, and developing innovative approaches such as telehealth and digital interventions. By working together, we can mitigate the impact of pandemics on childhood obesity and hypertension and pave the way for healthier futures for our children.

## Data Availability

More data are available on request from the corresponding authors.
